# A 5-Gene Signature Is Closely Related to Tumor Immune Microenvironment and Predicts the Prognosis of Patients with Non-Small Cell Lung Cancer

**DOI:** 10.1155/2020/2147397

**Published:** 2020-01-09

**Authors:** Jia Li, Huiyu Wang, Zhaoyan Li, Chenyue Zhang, Chenxing Zhang, Cheng Li, Haining Yu, Haiyong Wang

**Affiliations:** ^1^Department of Oncology, Longhua Hospital, Shanghai University of Traditional Chinese Medicine, Shanghai 200032, China; ^2^Institute of Physiological Chemistry and Pathobiochemistry, University of Muenster, Münster 48149, Germany; ^3^Department of Integrative Oncology, Fudan University Shanghai Cancer Center, Shanghai 200032, China; ^4^Department of Nephrology, Shanghai Children's Medical Center, Shanghai Jiao Tong University School of Medicine, Shanghai 200127, China; ^5^School of Health Care Management, Shandong University, Key Laboratory of Health Economics and Policy Research, Jinan 250100, China; ^6^Department of Internal Medicine-Oncology, Shandong Cancer Hospital and Institute, Shandong First Medical University and Shandong Academy of Medical Sciences, Jinan 250117, China

## Abstract

**Purpose:**

Establishing prognostic gene signature to predict clinical outcomes and guide individualized adjuvant therapy is necessary. Here, we aim to establish the prognostic efficacy of a gene signature that is closely related to tumor immune microenvironment (TIME).

**Methods and Results:**

There are 13,035 gene expression profiles from 130 tumor samples of the non-small cell lung cancer (NSCLC) in the data set GSE103584. A 5-gene signature was identified by using univariate survival analysis and Least Absolute Shrinkage and Selection Operator (LASSO) to build risk models. Then, we used the CIBERSORT method to quantify the relative levels of different immune cell types in complex gene expression mixtures. It was found that the ratio of dendritic cells (DCs) activated and mast cells (MCs) resting in the low-risk group was higher than that in the high-risk group, and the difference was statistically significant (*P* < 0.001 and *P*=0.03). Pathway enrichment results which were obtained by performing Gene Set Variation Analysis (GSVA) showed that the high-risk group identified by the 5-gene signature had metastatic-related gene expression, resulting in lower survival rates. Kaplan–Meier survival results showed that patients of the high-risk group had shorter disease-free survival (DFS) and overall survival (OS) than those of the low-risk group in the training set (*P*=0.0012 and *P* < 0.001). The sensitivity and specificity of the gene signature were better and more sensitive to prognosis than TNM (tumor/lymph node/metastasis) staging, in spite of being not statistically significant (*P*=0.154). Furthermore, Kaplan–Meier survival showed that patients of the high-risk group had shorter OS and PFS than those of the low-risk group (*P*=0.0035, *P* < 0.001, and *P* < 0.001) in the validating set (GSE31210, GSE41271, and TCGA). At last, univariate and multivariate Cox proportional hazard regression analyses were used to evaluate independent prognostic factors associated with survival, and the gene signature, lymphovascular invasion, pleural invasion, chemotherapy, and radiation were employed as covariates. The 5-gene signature was identified as an independent predictor of patient survival in the presence of clinical parameters in univariate and multivariate analyses (*P* < 0.001) (hazard ratio (HR): 3.93, 95% confidence interval CI (2.17–7.1), *P*=0.001, (HR) 5.18, 95% CI (2.6995–9.945), *P* < 0.001), respectively. Our 5-gene signature was also related to EGFR mutations (*P*=0.0111), and EGFR mutations were mainly enriched in low-risk group, indicating that EGFR mutations affect the survival rate of patients.

**Conclusion:**

The 5-gene signature is a powerful and independent predictor that could predict the prognosis of NSCLC patients. In addition, our gene signature is correlated with TIME parameters, such as DCs activated and MCs resting. Our findings suggest that the 5-gene signature closely related to TIME could predict the prognosis of NSCLC patients and provide some reference for immunotherapy.

## 1. Introduction

Lung cancer remains the leading cause of cancer morbidity and mortality, with 2.1 million new lung cancer cases and 1.8 million deaths expected in 2018 [1]. NSCLC accounts for up to 85% of all lung cancers and mainly comprises adenocarcinoma (65%) and squamous cell carcinoma (30%) histologies [2]. In the past few years, although molecular diagnostics and new treatments (targeted therapy, immunotherapy, etc.) have made much progress and the 5-year survival rate of most patients has increased slightly, the overall prospects have not been very large [3, 4].

The current TNM staging system is the best predictor of prognosis and the standard for guiding NSCLC treatment decisions [5]. However, due to the heterogeneity of the tumor itself and the complexity of the pathogenesis, even patients with the same TNM stage and treatment may exhibit various clinical outcomes [6]. Through microarray gene expression profiling to analyze and screen gene expression characteristics and establish a prognostic gene signature, it is better to predict clinical outcomes and guide the adjuvant treatment of individual patients than TNM staging. So far, several studies based on gene expression signatures have been shown to classify various cancer patients into different prognostic groups with different clinical characteristics [7–11]. However, the gene signatures closely related to TIME have not been found in NSCLC.

The type, density, and location of immune cells in the tumor microenvironment play an important role in the development of the disease [12]. Therefore, immunological structures based on the tumor microenvironment should be used as a separate component in the classification system [13]. Incorporating TIME parameters into gene signature will be more conducive to individualized treatment options [14]. However, regardless of the single monoclonal antibody immunohistochemistry technique or the flow cytometry of multiple antibodies, consistent and accurate data on immune cell composition were not obtained [15–20]. Therefore, the exact immune cell content in different tumors of NSCLC remains accurately undetermined. Several reports indicated that the relative levels of distinct immune cell types by the analytical platform CIBERSORT could estimate the immune cell composition in a tumor [21–23].

In this study, we used downloaded gene expression data and identified a 5-gene signature using univariate survival analysis and LASSO to distinguish between two prognostic groups (low and high risk). Then, we used the CIBERSORT method to quantify the relative levels of different immune cell types in complex gene expression mixtures. Furthermore, the validity and reliability of the 5-gene signature were further verified. Our findings suggest that the 5-gene signature closely related to TIME could predict the prognosis of lung cancer patients and provide some reference for immunotherapy.

## 2. Materials and Methods

### 2.1. Data Source and Processing

Gene expression profiling data of NSCLC patients were downloaded from Gene Expression Omnibus datasets (GEO; GSE103584, GSE31210, GSE41271) and the Cancer Genome Atlas (TCGA, https://tcga-data.nci.nih.gov/tcga/). Microarray analysis of 130 NSCLC patients in GSE103584 is based on Cancer SCAN panel [24]. The dataset GSE103584 was used as a training set for model construction, and data in GSE31210 [10], GSE41271 [25], and TCGA were applied to verify the validity of the model.

### 2.2. Screening for Prognosis-Related Genes and Building Risk Models

The LASSO was a better high-dimensional regression classifier and was used to select the key genes influencing patient outcomes [26]. The LASSO 1000 iterations were performed using the publicly available R package glmnet [27]. Multiple genomes containing the optimal solution were received after multiple dimensionality reduction. At the same time, for the stability and accuracy of the results, a random sampling method of leave-one-out cross validation (LOOCV) was used to select a set of genes to construct a prognostic model [26].

According to the selected genetic model, a risk formula of risk score was constructed to evaluate the high-risk and low-risk groups. The formula for obtaining the score is Σ_i_*ω*_i_*χ*_i_, where *ω*_i_ and *χ*_i_ are the coefficients and expressed value of each gene. The risk score for each sample in the data in the training set was calculated according to the formula, and the best cutoff value was generated using X-tile plots [28]. This threshold was set to classify patients: higher than the best cutoff for the low-risk group and lower than the risk score for the high-risk group.

### 2.3. Estimating the Composition of Immune Cells

To estimate the immune cell composition in the sample, the analytical platform CIBERSORT (https://cibersort.stanford.edu/) was used to quantify the relative levels of distinct immune cell types within a complex gene expression mixture [29]. The analysis was performed with an arrangement of 100 default statistical parameters. The activation and quiescence state of the same type of immune cells were analyzed as a whole. CIBERSORT's deconvolution of gene expression data provides valuable information about the composition of immune cells in a sample.

### 2.4. Analyzing Pathways with Differential Enrichment

GSVA, a pathway enrichment method that estimated variation of pathway activity over a sample population, was used to analyze changes in a pathway in each sample. GSVA was an open-source software package for R which forms part of the Bioconductor project and could be downloaded at http://www.bioconductor.org [30].

The prediction of the pathway under different disease states was made by the signal value of the gene and the pathway in which the gene was located. Firstly, the enriched score value of each sample was predicted by the signal value of the gene, and then the enrichment difference between the two groups was calculated, and the pathway with differential enrichment in the two groups was obtained. The screening standard *P* < 0.05, and the FDR < 0.05.

### 2.5. Validation of the Validity and Reliability

Univariate survival analysis of the gene signature was assessed by using survival in R language (*P* < 0.05) [31]. Then survival receiver operating characteristic curve (ROC) was used to complete the area under the curve (AUC) of 5-gene signature and TNM classification [32]. External data from GSE31210, GSE41271, and TCGA were applied to verify the reliability of the risk model's impact on the prognosis of the patients.

Fisher exact was used to assess the correlation between different gene mutation types and risk models. The univariate and multivariate Cox proportional hazard regression analyses were used to evaluate independent prognostic factors associated with survival. Risk model, lymphovascular invasion, pleural invasion, chemotherapy, and radiation were employed as covariates.

## 3. Result

### 3.1. Screening Genes Associated with Prognosis and Building Risk Models

**Figure 1:**
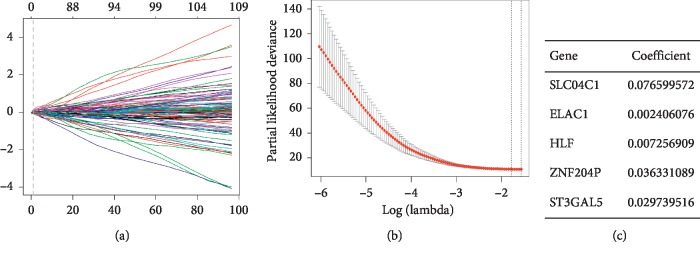
Screening genes associated with prognosis and building risk models. (a) Trend graph of LASSO coefficients. (b) Partial likelihood deviation map. (c) The name and coefficient of the 5-gene signature closely related to the immune system.

There are 13,035 gene expression profiles from 130 tumor samples in the data set GSE103584 (Supplementary [Supplementary-material supplementary-material-1]). First, the data of GSE103584 was processed uniformly, and then the genes detected in more than 50% of the samples were screened out and normalized. We applied the LASSO Cox regression model to predict and analyze the genes most relevant to prognosis in the 130 sample data. A random sampling method of 10-cross validation was used to construct a prognostic model containing five genes (Figure 1(a)). Through calculation and verification, it is found that the model constructed by 5 genes has the lowest error rate (Figure 1(b)).  Figure 1(c) shows the specific information and coefficients of the five genes. Characteristics of the patient in the training set (GSE103584) are given in Table 1.

### 3.2. Estimating the Composition of Immune Cells

**Figure 2:**
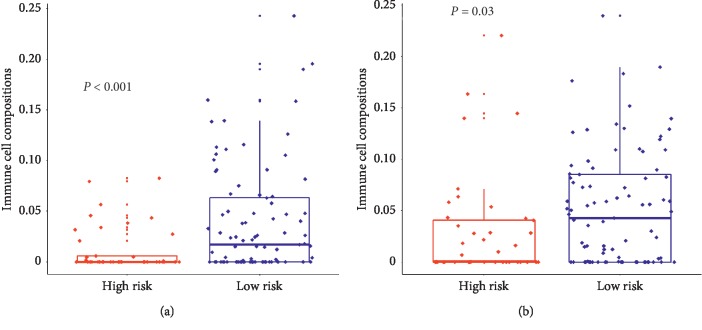
Estimating the composition of immune cells. (a) The ratio of dendritic cells activated in the high-risk and low-risk groups. (b) The ratio of mast cells resting in the high-risk and low-risk groups.

We used CIBERSORT to estimate the immune cell composition of 130 samples and quantify the relative levels of different cell types in a mixed cell population. All results were normalized to proportions by cell type (Supplementary [Supplementary-material supplementary-material-1]). As shown in Figures 2(a) and 2(b), we compared different types of cells in the low-risk group and the high-risk group. It was found that the ratio of dendritic cells activated and mast cells resting in the low-risk group was higher than that in the high-risk group, and the difference was statistically significant (*P* < 0.001 and *P*=0.03). The results suggested that the immune cells in the low-risk group were better activated.

### 3.3. Analysis of Differential Pathways

**Figure 3:**
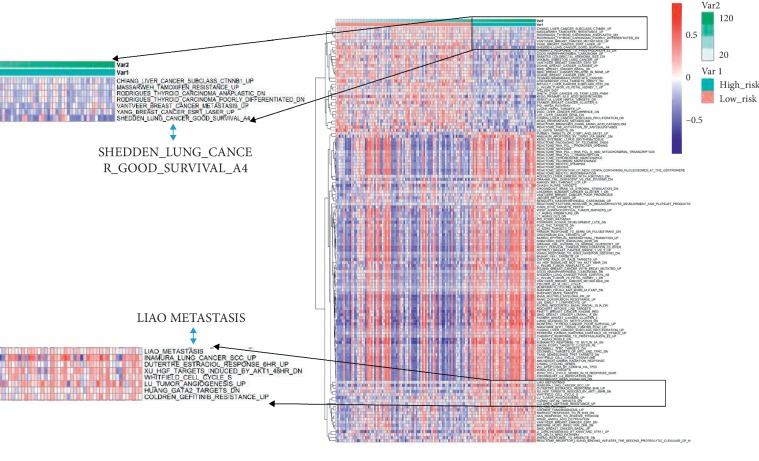
The changes in the pathways of 130 samples in the low-risk and high-risk groups.

By performing GSVA analysis on the differential genes of the low-risk group and the high-risk group, the changes in the relevant pathways in different states were obtained.  Figure 3 shows the changes in the pathways of 130 samples in the low-risk and high-risk groups. The result of the enrichment is SHEDDEN_LUNG_CANCER_GOOD_SURVIAL_A4, indicating that the prognostic grouping of the data is consistent with other data. LIAO_METASTASIS is gradually increasing in the low-risk group and the high-risk group, indicating high meta-expression of metastasis-related genes in the high-risk group.

### 3.4. Validation of the Validity and Reliability

**Figure 4:**
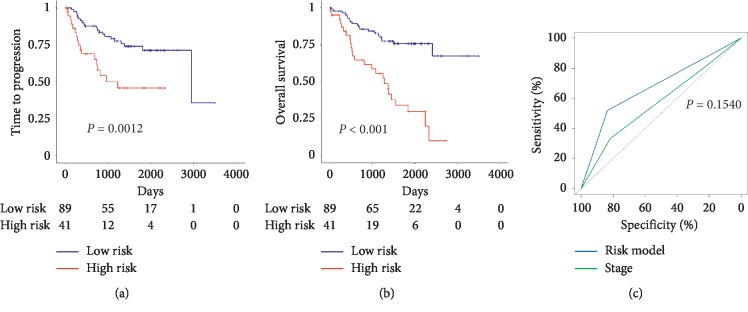
Kaplan–Meier survival curves and ROC curves in the training set. (a) Kaplan–Meier survival curves for relapse-free survival in the training set. (b) Kaplan–Meier survival curves for overall survival in the training set. (c) ROC curves of the risk model and TNM staging in the training set.

Survival analysis in R language pack was applied to examine the effects of different groups on the prognosis of NSCLC. Kaplan–Meier survival curves for relapse-free survival indicated the probability of recurrence in the high-risk group and the low-risk group. The results showed that patients in the high-risk group had shorter disease progression times than those in the low-risk group (Figure 4(a), *P*=0.0012). Kaplan–Meier survival curves for overall survival were used to represent the survival probabilities of the high-risk group and the low-risk group. The results showed that patients in the high-risk group had shorter overall survival than patients in the low-risk group (Figure 4(b), *P* < 0.001).

To further validate the accuracy of the risk prediction model, we established a ROC plot of the hazard model and TNM staging. As shown in Figure 4(c), we found that risk prediction models could be more sensitive to prognosis than TNM staging, in spite of being not statistically significant (*P*=0.154).

**Figure 5:**
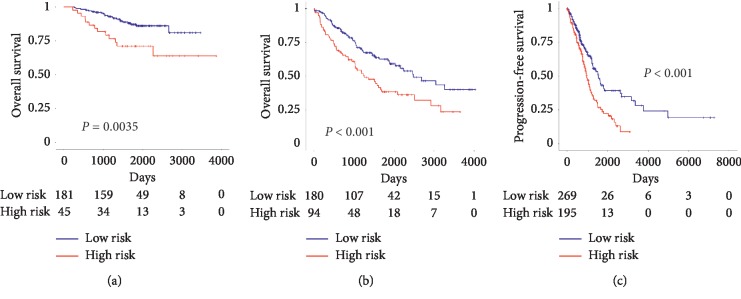
Kaplan–Meier survival curves for overall survival and progression-free survival in the validating set. Kaplan–Meier survival curves for overall survival in the (a) GSE31210 set, (b) GSE41271 set, and (c) TCGA.

Furthermore, external data from GSE31210, GSE41271, and TCGA were applied as a validating set to verify the validity and reliability of the 5-gene signature impact on the prognosis of the patients. Kaplan–Meier survival showed that patients in the high-risk group had shorter overall survival than patients in the low-risk group (Figure 5(a), *P*=0.0035 and Figure 5(b), *P* < 0.001) and patients in the high-risk group had shorter progression-free survival than those in the low-risk group (Figure 5(c), *P* < 0.001).

### 3.5. Correlation with Mutant Genes and Clinical Information

By observing the correlation between the predicted risk model and different mutant genes, we found that EGFR mutations were related to the risk model grouping (*P*=0.011), and EGFR mutations were mainly enriched in low-risk, indicating that EGFR mutations affect the survival rate of patients (Table 2). However, there was no correlation between ALK and KRAS gene mutations and risk models (*P* > 0.05). The univariate and multivariate Cox proportional hazard regression analyses were used to evaluate independent prognostic factors associated with survival. Risk model, lymphovascular invasion, pleural invasion, chemotherapy, and radiation were employed as covariates. It was found that the risk model constructed by the 5-gene signature was an independent risk factor for prognosis (Table 3, *P* < 0.001).

## 4. Discussion

Based on gene expression data and survival analysis techniques, we screened a 5-gene signature for predicting the prognosis of NSCLC patients. That is, differential expressions of 5 genes among Solute carrier organic anion transporter family member 4C1(SLCO4C1), ElaC ribonucleaseZ1(ELAC1), Hepatic leukemia factor (HLF), Zinc finger protein 204, pseudogene (ZNF204P), and ST3 beta-galactoside alpha-2,3-sialyltransferase 5 (ST3GAL5) will influence progression-free survival and survival time of NSCLC patients. External data from GSE31210, GSE41271, and TCGA were applied to verify the reliability of the 5-gene signature impact on the prognosis of the patients. To further validate the accuracy of the 5-gene signature, we established a ROC map of the hazard model and TNM staging. The sensitivity and specificity of the gene signature were better and more sensitive to prognosis than TNM staging, in spite of being not statistically significant (*P*=0.154).

We not only confirmed the stability and accuracy of the 5-gene signature, but also found it closely related to other clinical information. The changes in the relevant pathways in the differential genes of the low-risk group and the high-risk group were obtained by performing GSVA analysis. The results showed that the high-risk group identified by 5-gene signature had metastatic-related gene expression, resulting in lower survival rates. Our 5-gene signature was also related to EGFR mutations (*P*=0.011), and EGFR mutations were mainly enriched in the low-risk group, indicating that EGFR mutations affect the survival rate of patients. The univariate and multivariate COX regression model analysis was used to analyze the correlation between the 5-gene signature and other clinical factors. The 5-gene signature is an independent risk factor for prognosis (*P* < 0.001). These results suggest that our characteristics may contribute to clinical management.

Infiltrating immune cells are an integral component of the tumor microenvironment and play an important role in increasing the effectiveness of immunotherapy [33]. This infiltrating immune cell is usually a heterogeneous mixture of immune cells, including cell types associated with activity and inhibition [34]. Because of the need for different types and subtypes of TIME to be identified in the immunotherapy of tumors, their characteristics and differences are identified. In order to make substantial progress, bioinformatics techniques are used to assess the composition, functional status, and cellular localization of immune cells. Based on the gene signature, a more precise classification of patients based on their TIME will better observe overall survival and response to immunotherapeutic agents.

More importantly, we found that the 5-gene signature is closely related to TIME parameters. The success of cancer immunotherapy has revolutionized cancer treatment and has used TIME parameters (immune cell composition and proportion) as predictive immunotherapy markers [12]. Detailed characterization of immune cell composition in tumors may be the basis for determining the prognostic and predictive biomarkers of immunotherapy. Dendritic cells (DCs) are one of the core components of the immune system responsible for initiating an adaptive immune response that penetrates tumors and processes and presents tumor-derived antigens to naive T cells [35]. DC plays a key role in eliciting antitumor T cell immunity and thus represents the primary therapeutic target for cancer immunotherapy [36, 37]. Mast cells (MC) are thought to be involved in the regulation of innate and adaptive immune responses [38]. Furthermore, it is now recognized that MC is not only used as an effector cell but also induces T cell activation, recruitment, proliferation, and cytokine secretion in an antigen-dependent manner and affects regulatory T cells [39]. At present, it is increasingly found that mast cells play an important role in antitumor immunity [40]. We used CIBERSORT to estimate the immune cell composition of 130 samples to quantify the relative levels of different cell types in a mixed cell population and compared different types of cells in the low-risk group and the high-risk group. It was found that the ratio of dendritic cells activated and mast cells resting in the low-risk group was higher than that in the high-risk group, and the difference was statistically significant (*P* < 0.001 and *P*=0.03). The results suggested that the presence of immune cells was better activated and the prognosis was better in the low-risk group. In summary, the 5-gene signature closely related to TIME parameters could predict the prognosis of lung cancer patients and provide some reference for immunotherapy.

Notably, among the 5-gene signature, only the gene HLF is involved in tumor immunity and the gene ST3GAL5 is involved in tumor invasion, migration, and proliferation. There are two other genes (SLCO4C1, ELAC1) that may have a relationship with the development of tumors, but there is no clear report. The ZNF204P gene has not been reported. SLCO4C1 is a key tumor suppressor gene in head and neck cancer that can be inactivated by “larger promoter” methylation and somatic mutations [41]. Overexpression of the human kidney-specific organic anion transporter SLCO4C1 in rat kidneys reduces hypertension, cardiac hypertrophy, and inflammation in renal failure [42]. Hepatic leukemia factor (HLF) is a critical transcription factor that plays an important regulatory role in many cancers, especially leukemia [43, 44] and may be involved in therapeutically induced immunogenic cell death [45]. HLF is a gene involved in the transformation from E1 to E2, and its inhibition can produce a more immunogenic microenvironment [46]. Overexpression of ST3GAL5 significantly promoted the proliferation and invasion of hepatoma cells. In contrast, knockdown of ST3GAL5 inhibited proliferation and metastasis of hepatoma cells [47]. This indicates that ST3GAL5 is closely related to the invasion and metastasis of liver cancer. In addition, ST3GAL5 has been reported to be positively associated with high risk of childhood acute leukemia and is associated with multidrug resistance in human acute myeloid leukemia, indicating the role of ST3GAL5 in cancer development and progression [48, 49]. ELAC1 appears to correspond to the C-terminal half of 3′tRNase from ELAC2 and it was found that ELAC1 also has 3′-tRNase activity, possibly encoding a candidate prostate cancer susceptibility gene for tRNA 3′ processing endoribonucleases [50]. From the above results, we can see that our gene signature not only identifies new promising biomarkers but also may provide a direction for the study of TIME mechanisms.

Here, we identify that the 5-gene signature is a powerful and independent predictor that could predict the prognosis of lung cancer patients. In addition, our gene signature is correlated with TIME parameters, such as DCs activated and MCs resting. Our findings suggest that the 5-gene signature closely related to TIME could predict the prognosis of lung cancer patients and provide some reference for immunotherapy.

## Figures and Tables

**Table 1:** Clinicopathological characteristics of NSCLC patients in the training set.

Variables	Number	%
Age		
<65	37	28.5
≥65	93	71.5
Sex		
Female	34	26.2
Male	96	73.8
Histology		
Adenocarcinoma	96	73.8
Squamous	31	23.8
Other	3	2.3
T stage		
Tis	5	3.8
T1	53	40.8
T2	49	37.7
T3	16	12.3
T4	7	5.4
N stage		
N0	104	80
N1	12	9.2
N2	14	10.8
Radiation		
Yes	14	10.8
No	116	89.2
Chemotherapy		
Yes	37	28.5
No	93	71.5
EGFR status		
Yes	19	14.6
No	82	63.1
Unknown	29	22.3
ALK status		
Yes	2	1.5
No	97	74.6
Unknown	31	23.8
KRAS status		
Yes	24	18.5
No	77	59.2
Unknown	29	22.3

**Table 2:** The correlation between the 5-gene signature and different mutant genes.

Variables	Low risk	High risk	*P*
EGFR status			0.0112
Yes	18	1	
No	54	28	
ALK status			1
Yes	2	0	
No	57	20	
KRAS status			0.7944
Yes	17	7	
No	68	29	

**Table 3:** The univariate and multivariate Cox proportional hazard regression analyses between the 5-gene signature and other clinical factors of NSCLC patients.

Variables	Univariable analysis				Multivariable analysis			
	HR	Lower	Higher	*P*	HR	Lower	Higher	*P*
5-gene signature (high vs. low)	3.93	2.17	7.1	0	5.18	2.6995	9.945	<0.001
Lymphovascular invasion (yes vs. no)	1.37	0.58	3.26	0.476	1.03	0.4226	2.514	0.947
Pleural invasion (yes vs. no)	1.15	0.6	2.2	0.679	1.38	0.6977	2.745	0.352
Chemotherapy (yes vs. no)	1.1	0.59	2.05	0.76	0.94	0.4066	2.168	0.883
Radiation (yes vs. no)	1.26	0.56	2.84	0.579	1.42	0.4903	4.107	0.519

## Data Availability

We declared that materials described in the manuscript, including all relevant raw data, will be freely available to any scientist wishing to use them for noncommercial purposes, without breaching participant confidentiality.

## Supplementary Materials

Supplementary Materials Supplemental material 1: 13,035 gene expression profiles from 130 tumor samples in data set GSE103584. We used CBERSORT to estimate the immune cell composition of 130 samples and quantify the relative levels of different cell types in a mixed cell population. Supplementary material 2: the standardized relative proportion of all cell types.
